# ^1^H, ^13^C and ^15^N resonance assignment of backbone and IVL-methyl side chain of the S135A mutant NS3pro/NS2B protein of Dengue II virus reveals unique secondary structure features in solution

**DOI:** 10.1007/s12104-022-10071-w

**Published:** 2022-02-12

**Authors:** Peter Agback, Dmitry M. Lesovoy, Xiao Han, Renhua Sun, Tatyana Sandalova, Tatiana Agback, Adnane Achour, Vladislav Yu. Orekhov

**Affiliations:** 1 Department of Molecular Sciences, Swedish University of Agricultural Sciences, PO Box 7015, 750 07 Uppsala, Sweden; 2 Shemyakin-Ovchinnikov Institute of Bioorganic Chemistry RA, 117997 Moscow, Russia; 3 Science for Life Laboratory, Department of Medicine, Solna, Karolinska Institute, and Division of Infectious Diseases, Karolinska University Hospital, SE‑171 76 Stockholm, Sweden; 4 Department of Chemistry and Molecular Biology, University of Gothenburg, Box 465, 40530 Gothenburg, Sweden

**Keywords:** Dengue 2 virus, NS3 protease, Flavivirus protease, NMR chemical shifts assignment, Methyl assignment, Backbone dynamics

## Abstract

The serotype II Dengue (DENV 2) virus is the most prevalent of all four known serotypes. Herein, we present nearly complete ^1^H, ^15^N, and ^13^C backbone and ^1^H, ^13^C isoleucine, valine, and leucine methyl resonance assignment of the apo S135A catalytically inactive variant of the DENV 2 protease enzyme folded as a tandem formed between the serine protease domain NS3pro and the cofactor NS2B, as well as the secondary structure prediction of this complex based on the assigned chemical shifts using the TALOS-N software. Our results provide a solid ground for future elucidation of the structure and dynamic of the apo NS3pro/NS2B complex, key for adequate development of inhibitors, and a thorough molecular understanding of their function(s).

**Supplementary Information:**

The online version contains supplementary material available at 10.1007/s12104-022-10071-w.

## Biological context

Serine proteases of Dengue virus (DENV 1–4) have been studied intensively due to their critical roles in polyprotein maturation and viral infectivity. It has been demonstrated that the DENV-2 multifunctional protein NS3 comprises a serine protease domain (NS3pro) that requires the conserved hydrophilic domain of NS2B as a cofactor for adequate protease activity, resulting in the cleavage of the polyprotein precursor at sites comprising two basic amino acids (Yusof et al. 2000). In vitro studies have also previously revealed that a hydrophilic NS2B-derived 40 amino acid residues-long segment is sufficient to form an active NS3pro/NS2B complex (Falgout et al. 1991). It should be noted that, at a structural level, the cofactor NS2B is presumed to prevail in two conformations; either in an ‘open state’ in which it folds away from the active site of NS3 or in ‘closed’ state in which the NS2B C-terminus associates with NS3pro, folding over the active site.

The complexity of the ‘open’ and ‘closed’ conformations of the DENV protease and its structure has been a controversial and highly discussed topic during recent years. The dominance of one vs another conformation of NS2B was associated with the type of expression construct, interaction with inhibitor or different experimental conditions, such as buffer pH and salt concentration. Moreover, it was conventionally postulated that since the C-terminal part of NS2B is essential for proteolytic activity in both the DENV (Erbel et al. 2006; Niyomrattanakit et al. 2004; Phong et al. 2011; Yusof et al. 2000) and the closely related West Nile virus (WNV) serine proteases (Radichev et al. 2008), the so called ‘closed’ conformation is the enzymatically active structure. Two type of constructs were generally used: (1) the ‘linked’ construct, in which the C-terminus of NS2B is covalently linked to the N-terminus of NS3 via the flexible linker Gly4-Ser-Gly4 (Leung et al. 2001), (2) the ‘unlinked’ construct (Kim et al. 2013; Woestenenk et al. 2017).

It has also generally been confirmed that DENV and WNV protease complexes with inhibitors preferably adopted the ‘closed’ conformation regardless if a ‘linked’ or ‘unlinked’ NS3pro/NS2B construct was used and if the studies were performed using NMR (Agback and Agback 2018; Agback et al. 2020; Chen et al. 2016; Cruz et al. 2014; Gibbs et al. 2018; Kim et al. 2013; Pilla et al. 2015; Woestenenk et al. 2017) or X-ray crystallography (Erbel et al. 2006; Noble et al. 2012).

The positions of NS2B relative to NS3pro in different types of ligand-free apo DENV and WNV NS3pro/NS2B complexes are differing depending on the constructs and structural methods used (X-ray crystallography or NMR). Indeed, the crystal structures of the ligand-free DENV1 (Chandramouli et al. 2010), DENV2 (Erbel et al. 2006) and DENV4 (Luo et al. 2008) serine proteases have been determined in complex with various lengths of the cofactor NS2B on ‘linked’ constructs. The cofactor NS2B takes an ‘open state’ conformation when in complex with DENV1 or DENV2 NS3pro, folding away from their active sites. In contrast to these crystal structures, NMR spectroscopy (Kim et al. 2013; Woestenenk et al. 2017) and paramagnetic labelling studies (Cruz et al. 2014; Pilla et al. 2015) demonstrated that NS2B predominantly adopts a ‘closed’ conformation in solution, even in the absence of substrate-like inhibitors. It has therefore been argued that the open state of ligand-free DENV NS3pro/NS2B complexes observed in crystal structures may be caused by crystal packing or interference of the linker. It is also worth mentioning that a recent analysis of the variations of the NS3pro/NS2B fold in flaviviral proteases, based on X-ray structures deposited in the PDB, allowed the authors to argue that the concept of ‘open/closed’ conformations is an oversimplified model which probably does not reflect the complex nature of these serine proteases (Behnam and Klein 2020). The same authors proposed that local and global conformational changes in the NS3 protease domain and the dynamics of the relative positions of NS2B to NS3pro should be considered in order to acquire a better understanding of the molecular basis underlying their function.

NMR spectroscopy is the primary method to elucidate the dynamic of proteins and its complexes in solution. Here we focused on the NS3pro/NS2B protease from DENV2, the most prevalent dengue virus serotype. To obtain a complete description of the catalytic mechanisms in DENV2, and potentially develop efficient inhibitors in a rational way based on unambiguous structural basis, it is in our opinion of considerable importance to (i) establish how domains in NS3pro and its cofactor NS2B potentially change orientation, and (ii) identify the intra domain structural perturbations that occur in response to ligand binding by the apo form of this enzyme. We believe that the inter domain structures of the apo DENV2 NS3pro/NS2Bcomplex in solution can be quite different compared to their relative positions within the asymmetric unit of crystals. To fulfill this task we previously performed an ^1^H, ^15^N, and ^13^C assignment of the backbone resonances for the ‘unlinked’ DENV2 NS3pro/NS2B complex bound to a boronic acid tetrapeptidic inhibitor, where all key amino acids in the catalytic triad and the oxyanion hole were successfully identified (BMRB code 26996) (Woestenenk et al. 2017). Although a dataset for the assigned backbone chemical shifts of the apo form of the DENV2 NS3pro/NS2B protease co-expressed in an ‘unlinked’ version is available (Kim et al. 2013), the derived secondary structure predicted from these chemical shifts are not fully consistent with the X-ray-based three-dimensional structure of the apo DENV2 ‘linked’ NS3pro/NS2B complex (Erbel et al. 2006), nor with the secondary structure of the ‘unlinked’ DENV2 NS3pro/NS2B in complex with the boronic acid tetrapeptidic inhibitor (Woestenenk et al. 2017). Besides these structural inconsistencies, several sequence differences between their and our constructs renders a thorough comparative analysis difficult with uncertain results.

Since the three-dimensional structure of the apo form of the DENV2 NS3pro/NS2B complex in solution remained missing, we initiated a detailed NMR investigation starting with the apo form of the S135A mutated protein variant. This mutation renders the protease inactive as the serine in the catalytic triad (H51-D75-S135) is changed into an alanine with minimal interference on the overall structure (Agback et al. 2020). Applying the target acquisition (TA) methodology (Isaksson et al. 2013; Jaravine et al. 2008), we performed near complete (> 95%) backbone ^1^HN, ^15^N, ^13^C^α^, ^13^CO, ^1^H^α^ and sidechain ^13^C^β^ chemical shift assignments of the DENV2 S135A NS3pro/NS2B complex. We also assigned the methyl resonances for the side chains of valine, leucine, and isoleucine residues. This resonance assignment will provide a crucial tool for mapping protein–protein interaction sites in DENV2 NS3pro/NS2B and for understanding how these binding events affect the structure and dynamics of the enzyme in order to modulate its role in substrate binding.

## Methods and experiments

### Expression constructs

The codon optimized cDNA encoding NS3pro/NS2B (strain TSV01) was synthesized (MWG Eurofins), flanked by NdeI and XhoI sites for subcloning into pET21b (Novagen). The NS2B construct (residues 47–95, amino acids 1394–1440 of the Dengue 2 polyprotein) and NS3pro (residues 1–185; amino acids 1476–1660 of the polyprotein) were generated as described (Woestenenk et al. 2017). Briefly NS2B was subcloned into pET21b using NdeI and BamHI sites. A His6 -thrombin protease cleavage site was introduced at the N-terminus of NS2B, generating His-thrombin-NS2B. A His6 tag was introduced at the N-terminus of NS3pro, and it was subcloned into pET21b using NdeI and XhoI sites. NS3pro-Ser135Ala mutation of active site residues was introduced using the QuikChange Lightning kit (Agilent). The correctness of the inserted DNA was verified by sequencing. Reagents were from Sigma (St. Louis, MO, USA) unless otherwise stated.

### Protein expression and purification for NMR studies

The NS2B and NS3pro constructs were transformed into *E. coli* T7 express competent cells and expressed separately in different isotopic labelling combinations in ^1^/^2^H, ^15^ N, ^12^/^13^C-labelled M9 medium. Chemicals for isotope labelling (ammonium chloride, ^15^N (99%), d-glucose, ^13^C (99%), deuterium oxide) were purchased from Cambridge Isotope Laboratories. Inc. Protein expression was induced for 4–5 h at 310 K by addition of β-d-1-thiogalactopyranoside (IPTG) to 1 mM final concentration. When the cell optical density at 600 nm (OD600) reached 0.9–1.0, cells were harvested by centrifugation at 6000 g.

A methyl protonated Ileδ1-[^13^CH_3_], Leu, Val-[^13^CH_3_/^12^CD_3_], U-[^15^N,^13^C,^2^H] sample of S135A NS3pro/NS2B was obtained as previously described (Tugarinov et al. 2006). The protein was expressed in 1 L of D_2_O M9 medium using 3 g/L of U-[^13^C,^2^H]-glucose (CIL, Andover, MA) as the main carbon source and 1 g/L of ^15^NH_4_Cl (CIL, Andover, MA) as the nitrogen source. One hour prior to induction, precursors were added to the growth medium as previously described (Tugarinov et al. 2006). For precursors, 70 mg/L alpha-ketobutyric acid, sodium salt (^13^C4, 98%, 3,3-^2^H, 98%) and 120 mg/L alpha-ketoisovaleric acid, sodium salt (1,2,3,4-^13^C4, 99%, 3, 4, 4, 4, -^2^H 97%) (CIL, Andover, MA) were used. Bacterial growth was continued for 2 h at 310 K and the cells were thereafter harvested by centrifugation.

Following in vitro refolding protocol (Woestenenk et al. 2017) and subsequent purification steps, the NS3pro/NS2B S135A mutant complex was concentrated to 0.4–0.8 mM for data acquisition in NMR buffer containing 20 mM deuterated MES pH 6.5, 100 mM NaCl, 5 mM CaCl_2_, 0.02% NaN_3_, 1 × cocktail (Halt™ Protease Inhibitor Cocktail, EDTA-free 100X, Thermo Scientific™), 10% D2O.

### NMR spectroscopy

#### Backbone ^1^H, ^15^N, ^13^C resonance assignment of DENV2 S135A NS3pro/NS2B using the Targeted Acquisition (TA) approach (Isaksson et al. 2013; Jaravine and Orekhov 2006; Jaravine et al. 2008; Unnerstale et al. 2016)

All NMR experiments for backbone resonance assignment were performed at 298 K either on an 800 MHz Bruker AVANCE III-HD spectrometer equipped with a 3 mm cryo-enhanced TCI probe or on a 600 MHz Bruker Avance III spectrometer equipped with a 5 mm cryo-enhanced QCI-P probe. 2D ^1^H-^15^N transverse relaxation optimized spectroscopy (TROSY) was used (Eletsky et al. 2001; Pervushin et al. 1997; Schulte-Herbruggen and Sorensen 2000). The TA procedure was implemented on an 800 MHz spectrometer using the iterative non-uniformly sampled (NUS) Best-TROSY experiments (Favier and Brutscher 2011) with deuterium decoupling: 3D HNCO, 3D HNCOCA, 3D HNCA, 3D HNCACO, 3D HNCOCACB and 3D HNCACB. The latter two experiments were optimized for detection of cross peaks for ^13^C^β^ nuclei. All spectra were recorded with an inter-scan delay of 0.5 s. The experimental parameters for TA acquisition in the 3D experiments are summarised in Table 1.

**Table 1:** List of acquisition parameters used for NMR experiments for assignment of the backbone resonances

Experiments	Maximum evolution time, (ms)/carrier frequency (ppm)/sweep width (ppm)			Scans	NUS points	NUS %	Time (h)
	F3	F2	F1				
^1^H-^15^N TROSY^a,c^	119.8(^1^H)/4.7/16.0	87.7(^15^N)/118.0/36.0	–	8	–	–	
3D Best-TROSY-HNCO^a^	159.9(^1^H)/4.7/16.0	21.6(^15^N)/118.0/36.0	34.8(^13^C)/172.0/14.0	4	1544	25	4.5
3D Best-TROSY-HN(CA)CO_2H^a,b^	106.5(^1^H)/4.7/16.0	21.6(^15^N)/118.0/36.0	34.8(^13^C)/172.0/14.0	8	2460	40	14.4
3D Best-TROSY-HNCA_2H^a,b^	106.5(^1^H)/4.7/16.0	21.6(^15^N)/118.0/36.0	12.9(^13^C)/52.0/30.0	4	980	20	2.9
3D Best-TROSY-HN(CO)CA_2H^a,b^	106.5(^1^H)/4.7/16.0	21.6(^15^N)/118.0/36.0	12.9(^13^C)/52.0/30.0	4	980	20	2.9
3D Best-TROSY-HNCACB_2H^a,b^	106.5(^1^H)/4.7/16.0	21.6(^15^N)/118.0/36.0	13.0(^13^C)/39.0/70.0	12	4620	40	40.6
3D Best-TROSY-HN(CO)CACB_2H^a,b^	106.5(^1^H)/4.7/16.0	21.6(^15^N)/118.0/36.0	13.0(^13^C)/39.0/70.0	8	4620	40	27.0
3D H(CC)(CO)NH^c^	106.5(^1^H)/4.67/16.0	12.2(^15^N)/118/40.0	5.8(^13^C)/39/80.0	32	525	25	20.5
3D ^1^H–^15^N NOESY^c^	106.5(^1^H)/4.67/16.0	12.3(^15^N)/118/40.0	6.6(^1^H)/4.67/16.0	8	–	–	42.0
3D ^1^H–^13^C NOESY^c^	113.6(^1^H)/4.67/15.0/	3.3(^13^C)/43.0/80.0	16.7(^1^H)/4.67/15.0	8	–	–	64.0

^a^Target Acquisition experiments performed on an 800 MHz spectrometer

^b^Experiments performed with deuterium decoupling

^c^Experiments performed on a 600 MHz spectrometer

In order to assign H^α^ proton resonances, additional ^1^H-^15^N-NOESY, ^1^H-^13^C- NOESY and H(CC)(CO)NH data were collected on a 600 MHz spectrometer (Table 1) (Kay et al. 1990, 1992; Schleucher et al. 1994). The combined 3D NUS NMR TA acquisition data were processed using the IST algorithm in the NUS module in TopSpin4.0.6.Analysis was performed manually in CcpNmr Analysis 2.2.2 (Vranken et al. 2005).

#### ^1^H, ^13^C ile, leu, val methyl resonances assignment of DENV2 NS3pro/NS2B S135A

NMR experiments for the assignment of the ^1^H, ^13^C methyl groups of Val, Leu, Ile amino acids were recorded on a 900 MHz Bruker AVANCE III-HD spectrometer equipped with a 5 mm cryo-enhanced TCI probe. The assignment was based on a set of 3D resonance experiments including HMCM(CGCB)CA and HMCM(CGCBCA)CO for Ile/Leu and HMCM(CB)CA and HMCM(CBCA)CO for Val amino acids. The pulse programs were identical to hmcmcbcagpwg3d and hmcmcbcacogpwg3d in Bruker TopSpin3.6 except that 2H decoupling (Tugarinov and Kay 2003) was applied and 1.8 ms IBurp1 pulse (from the Bruker TopSpin3.6 library) was used for selective inversion of CG2 of Ile. The experimental parameters for all 3D experiments are summarised in Table 2. The 3D NUS methyl related experiments were processed using NMRpipe (Delaglio et al. 1995) and the IST algorithm in mddnmr software (Kazimierczuk and Orekhov 2011; Mayzel et al. 2014). Decoupling of the homonuclear one-bond ^13^C-^13^C^β^ scalar coupling in the HMCM(CB)CA and HMCM(CGCB)CA experiments was performed by deconvolution (Kazimierczuk et al. 2020).

**Table 2:** List of acquisition parameters used for NMR experiments for assignment of the Ile, Leu and Val methyl resonances

Experiments	Maximum evolution time, (ms)/currier frequency (ppm)/sweep width (ppm)			Scans	NUS points	NUS %	Time (h)
	F3	F2	F1				
^1^H-^13^C HSQC^a^	92(^1^H)/4.7/13.0	27.6(^13^C)/20.0	–	8	–	–	
HMCM(CGCBCA)CO_2H^a,b^	87.4(^1^H)/4.7/13.0	15.7(^13^C)/16.0/16.0	32.2(^13^C)/173.0/11.0	16	1368	30	31.9
HMCM(CGCB)CA_2H^a,b^	87.4(^1^H)/4.7/13.0	15.7(^13^C)/16.0/16.0	35.3(^13^C)/39/20.0	16	993	10.9	22.7
HMCM(CBCA)CO_2H^a,^^c^	87.4(^1^H)/4.7/13.0	15.7(^13^C)/16.0/16.0	32.1(^13^C)/173.0/11	16	410	9.0	9.5
HMCM(CB)CA_2H^a,c^	87.4(^1^H)/4.7/13.0	15.7(^13^C)/16.0/16.0	35.3(^13^C)/39.0/20.0	16	1103	12.1	24.8

^a^Experiments on 900 MHz spectrometer

^b^Optimized for Ile and Leu

^c^Optimized for Val

The ^1^H, ^13^C and ^15^N chemical shifts were referenced to DSS-d6. The ^13^C and ^15^N chemical shifts were referenced indirectly.

#### Estimation of the secondary structure of DENV2 NS3pro/NS2B S135A

The chemical shifts for the NS3pro/NS2B S135A mutant were analyzed with the TALOS-N software (Shen and Bax 2013). As input for TALOS-N analysis, the experimentally derived chemical shifts of ^1^HN, ^15^N, ^13^C^α^, ^13^C^β^, ^13^C´ and ^1^H^α^ nuclei for every amino acid were used. In case of no chemical shift, TALOS-N uses a database of sequences to predict the secondary structure.

### Extent of assignments and data deposition

#### DENV2 S135A NS3pro/NS2B mutant backbone resonances assignment by NMR

Using an array of large (over 40 kDa) folded proteins (Unnerstale et al. 2016) and fully intrinsically disordered proteins (IDP) (Agback et al. 2019), we have recently demonstrated that the targeted acquisition (TA) method, which is based on incremental non-uniform sampling (NUS), can be reliably used to speed up (1) data acquisition (Table 1), (2) achieve the best optimal resolution in 3D experiments (Table 1) in indirect dimensions compared with conventional methods, and last but not least (3) facilitate the assignment procedure (Orekhov and Jaravine 2011). Essential features of TA methods are simultaneous co-processing with multidimensional decomposition (co-MDD) of all triple-resonance spectra (Jaravine et al. 2008), which can therefore be efficiently used by the automated assignment software FLYA (Schmidt and Guntert 2012). To the best of our knowledge, the TA assignment method has hitherto been used only for monomeric proteins. In this study we demonstrate that TA approaches can be successfully applied to the heterodimeric uniformly labelled DENV2 S135A NS3pro/NS2B ‘unlinked complex’. The assignment procedure was further facilitated by using separate ^13^C^15^N^2^H isotope labelling of NS3pro and NS2B, combined with unlabelled NS3pro and NS2B. This latter procedure has previously been described (Woestenenk et al. 2017).

**Fig. 1:**
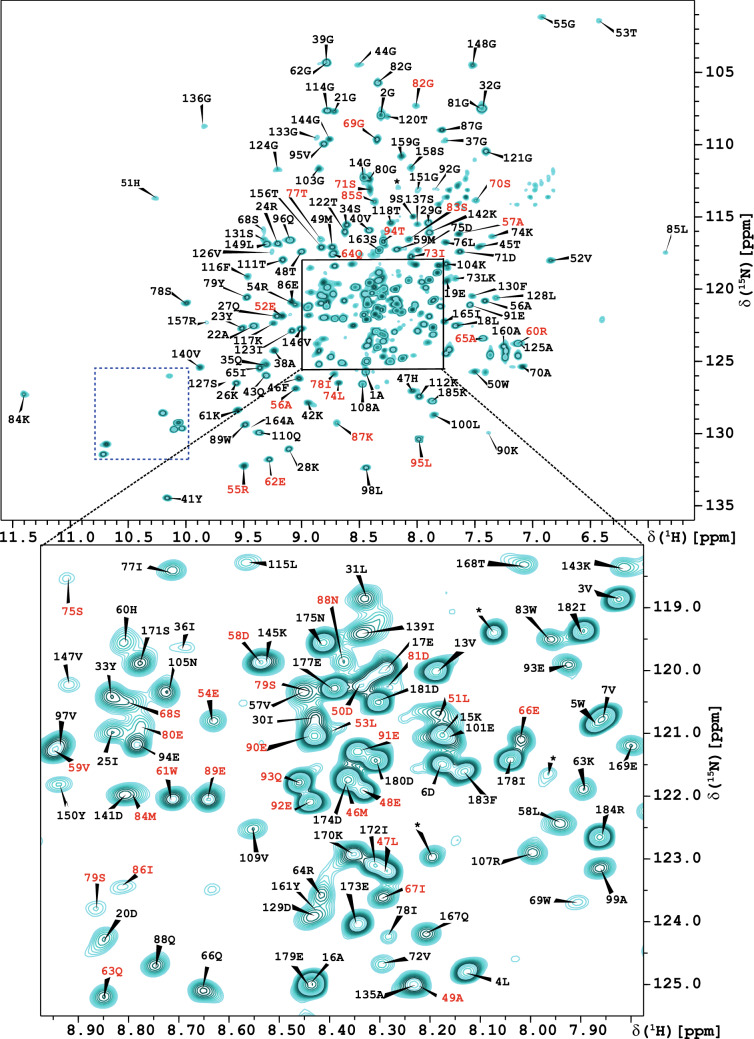
Annotated ^1^H,^15^N-HSQC spectrum of the DENV2 S135A NS3pro/NS2B complex. The spectrum of ^13^C,^15^N,^2^H-labelled S135A NS3pro/NS2B complex of 0.7 mM concentration in buffer 20 mM deuterated MES, 100 mM NaCl, 5 mM CaCl_2_, 0.02% NaN_3_, 1 × cocktail, pH 6.5, supplemented with 10% D_2_O, was acquired on an 800 MHz spectrometer at 298 K. The numbering of cross peaks was performed in accordance with the sequences of NS3pro (residues 1–185) and NS2B (residues 43–95) and colored in black and red respectively. A more detailed description of the central region is presented in the insert. Unidentified resonances are marked with asterisks (*). Peaks corresponding to the six (five belong to NS3pro and one to NS2B) tryptophan indole ring amide resonances and four N–H histidine aromatic resonances appear in the region of spectrum at ~ 10 ppm (^1^H) and ~ 129 ppm (^15^N), respectively, and are shown in a dashed box on the left part of the spectrum

**Fig. 2:**
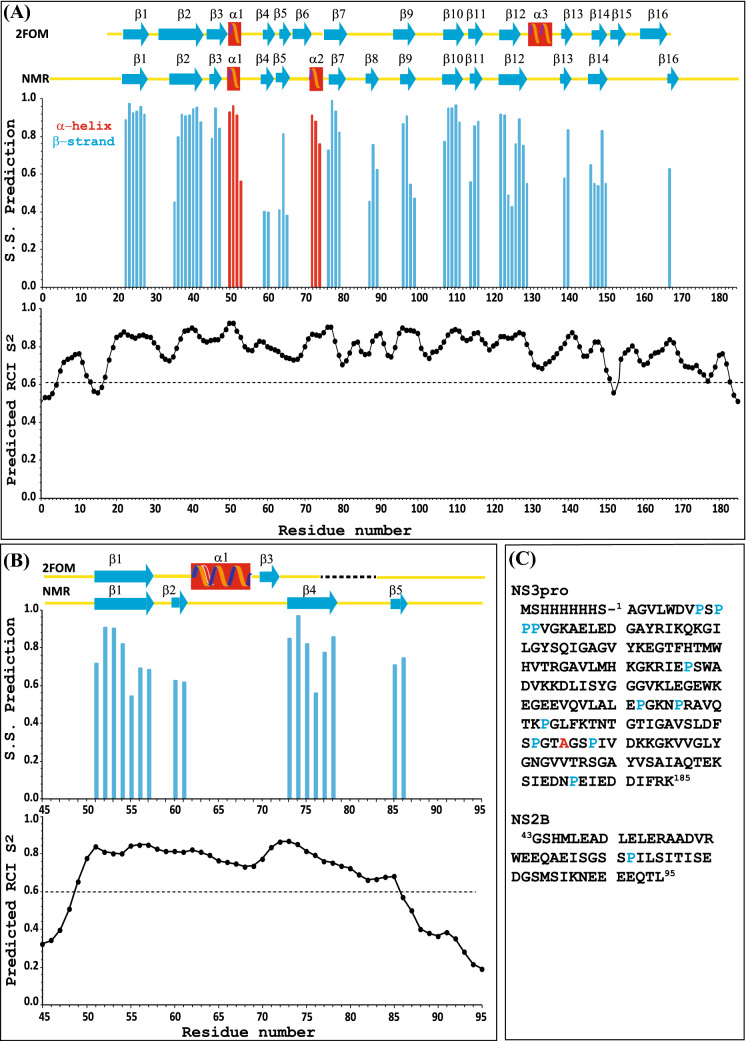
Secondary structure and comparison with previously determined secondary structures of the DENV2 NS3pro/NS2B complex. Secondary structure prediction using TALOS-N software (Shen and Bax 2013) based on the assigned chemical shifts of the NS3pro (Panel A) and NS2B (Panel B) of the DENV 2 S135A proteins reveals probabilities for α-helix (red) and β-strand (blue) secondary structures (SS Prediction) and RCI order parameter, S^2^, (Berjanskii and Wishart 2005) extracted by TALOS-N for each NH of the backbone. The yellow lines correspond to loop segments in the protein complex. The secondary structures along the sequence are indicated at the top of Panels **A** and **B**, where red squares correspond to α-helices and blue arrows to β-strands. The secondary structures derived from the previously determined crystal structure of the DENV 2 NS3/NS2B complex (residues 18–165 and 45–95 for NS3pro and NS2B respectively, PDB: 2FOM) presented on top of (**A**) and (**B**) panels according to UCSF Chimera (Pettersen et al. 2004). In Panel C the sequences of NS3pro and NS2B used within the present study are displayed with corresponding numbering. The mutation S135A is indicated in red. Proline residues are highlighted in blue

NS3pro is composed of 185 residues, plus a 9 amino acids long tag while the NS2B cofactor domain is composed of 53 residues (Fig. 2C). The NS3pro and NS2B sequences include 11 and 1 proline residues, respectively. Therefore, we expected to observe 226 peaks in the ^1^H, ^15^N HSQC spectrum (Fig. 1). Analysis of the 3D NMR data from the DENV2 NS3pro/NS2B S135A mutated variant enabled the identification and unambiguous assignment of 165 amides (95%) for NS3pro (Fig. 1; cross peaks labelled in black) and 49 (94%) for NS2B (Fig. 1; cross peaks labelled in red) of the 226 expected peaks. For the NS3pro protease domain, 92% of ^15^N (including proline residues), 97% of ^13^C^α^, 93% of ^13^C^β^, 92% of ^13^C´ and 76% of all H^α^ were assigned. For the cofactor NS2B (residues 43–95) 94% of ^15^N, 100% of ^13^C^α^, 96% of ^13^C^β^, 98% of ^13^C´ and 66% of all H^α^ were assigned. The chemical shift assignments have been deposited in the Biological Magnetic Resonance Data Bank (BMRB; http://www.bmrb.wisc.edu/) with accession code 51149.

#### Secondary structure of the DENV2 NS3pro/NS2BS 135A complex

The accuracy of the secondary structure analysis performed by TALOS-N based on chemical shifts (CS) increases with the number of CS used. Thus, in addition to CS of the ^1^HN, ^15^ N, ^13^C^α^, ^13^C^β^ and ^13^C´ nuclei we used the fully protonated ^15^N, ^13^C-labelled NS3pro/NS2B complex to also determine the CS of ^1^H^α^. Despite the large molecular weight of this complex, we were able to assign 74% of ^1^H^α^ using NOESY type experiments. The secondary structure analyses for NS3pro and the cofactor NS2B are presented in Fig. 2. Additionally, the TALOS-N-predicted inter-β-strands interactions within the folded domain of the NS3pro/NS2B complex were validated through observation of NOE contacts formed between ^1^HN–^1^HN or ^1^H^α^–^1^HN protons (Wüthrich 1986) in the NOESY spectrum.

Comparison of the secondary structures of S135A NS3pro/NS2B, which were derived from the chemical shift data acquired in this study, and the previously determined crystal structure (pdb code 2FOM) (Erbel et al. 2006), reveals striking similarities between the stretches of residues 18–130 and 45–57 for NS3pro and NS2B, respectively. However, we also observed a few important differences that are especially evident at the C-terminal end of both NS2B and NS3pro. For NS3pro, structural features are lacking between residues 150 and 185, which stands in contrast with the previous results from the X-ray structure in which two short β strands were observed (Fig. 2A top). Additionally, the RCI order parameter S^2^ for NS3pro backbone amides, predicted by TALOS-N based on their chemical shifts, shows a gradually decreasing trend starting from residue 130 and onwards (Fig. 2A bottom), indicating that the region is less well-structured in solution. Note that there are significant dips in RCI S^2^ around residues 133 and 152 which the X-ray structure say belong to the α3 helix and β15 strand respectively both elements missing from the NMR derived secondary structure. For NS2B, there are basically no similarities after residue 57 between the X-ray-derived secondary structure and the solution NMR structure. Importantly, our analysis reveals (Fig. 2B top) that in the X-ray structure of the NS3/NS2B complex, α1-helix and β3-strands were described in the NS2B regions 62–69 and 70–72, respectively, while residues 73–95 were described as random coil. Instead, in our NMR structure (Fig. 2B top) these sections are replaced by the long β-strand: β4 which is six amino acids long. The β4 strand is ordered according to the predicted RCI S^2^ (Fig. 2B bottom) and most likely interacts with NS3pro. It should also be noted that the entire segment after residue 86 in NS2B is completely lacking structural elements with low RCI S^2^ values (Fig. 2B bottom).

The secondary structure data obtained in this study is not in agreement with the earlier published NMR results for the apo form of the DENV2 NS3pro/NS2B complex (BMRB 19080 for the chemical shifts) (Kim et al. 2013) (Fig. S1). Indeed, the NS3pro structure by Kim et al*.* is missing the short α helices that are observed in both our NMR analysis of the DENV2 S135A NS3pro/NS2BS complex and in the corresponding crystal structure (pdb code 2FOM). Additionally, the largest discrepancies between our secondary NMR structure and the one by Kim et al*.* are observed for the C-terminal ends of both NS2B and NS3pro. In the Kim et al*.* secondary structure for NS2B, amino acids in the region 83–95 are fully folded in a long β-strand which are found as disordered in the secondary structures derived from our NMR of the S135A NS3pro/NS2BS, except a possible short β5-strand, as well as from the X-ray structure (2FOM). Also, in contrast to our results, the C-terminal end of NS3pro by Kim et al*.* seems ordered and comprises several β strands. A possible explanation for these significant differences could be due to differences in protein sequences between our construct and the one used by Kim et al. (four residues in NS3pro and two in NS2B are differing between our constructs; Fig. S1), or differences in experimental conditions (Behnam and Klein 2020).

#### Assignment of ^1^H, ^13^C resonances for methyl ile, leu and val residues in the DENV2 S135A NS3pro/NS2B mutated variant

**Fig. 3:**
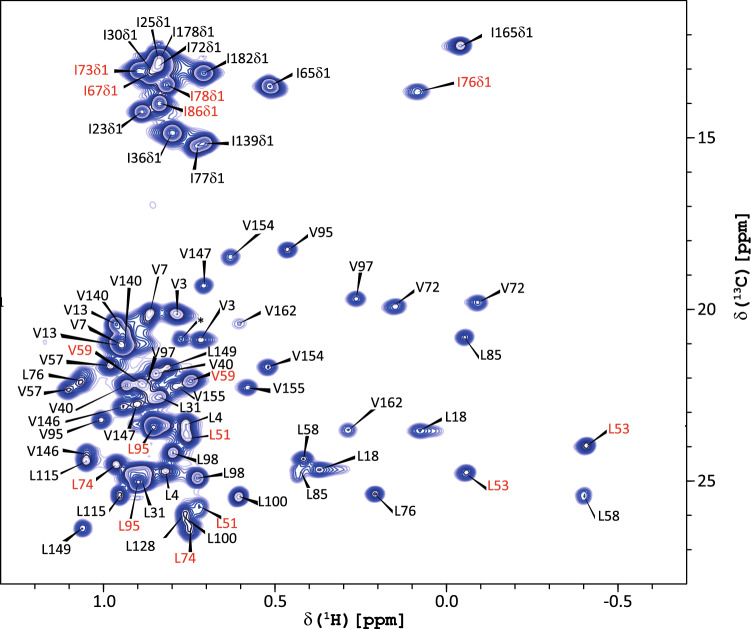
Annotated ^1^H, ^13^C-HSQC spectrum of the DENV2 S135A NS3pro/NS2B complex. The ^1^H–^13^C HSQC spectrum of the methyl labelled Ileδ1-[^13^CH_3_], Leu, Val-[^13^CH_3_/^12^CD_3_], U-[^15^N,^13^C,^2^H], DENV2 S135A NS3pro/NS2B mutated variant recorded at 900 MHz at 298 K is shown. Assignments are indicated in black for the NS3pro domain and in red for the NS2B cofactor

Knowledge of both backbone and side chain dynamics is important for a complete description of the dynamic and structural features of the DENV2 NS3pro/NS2B complex. Additionally, information retrieved from hydrophobic interactions between methyl groups could further highlight NS3pro/NS2B interactions. Our work provides the assignment of methyl ^1^H, ^13^C resonances of Ile, Leu, Val residues (Fig. 3), based on approaches described in (Tugarinov and Kay 2003). For NS2B, all isoleucine (5), valine (1) and leucine (4), besides the terminal residue L47, methyl groups are assigned. For NS3pro, 14 valine residues out of a total of 17 amino acids (the missing valine residues are V52, V109 and V126) were unambiguously assigned. Furthermore, all 11 leucine residues (except of one of the two L128 methyls) and all isoleucine were successfully assigned. The stereochemistry of the valine and leucine methyl groups are not defined in this study and will be addressed in the full structural and dynamic elucidation of the NS3pro/NS2B complex.

## Conclusion

In conclusion we present in this study the near complete ^15^N/^13^C/^1^H backbone and Ile/Leu/Val methyl resonance assignments for the apo form of the DENV2 S135A NS3pro/NS2B complex. Our results indicate that the C-terminal amino acid residues of both NS3pro (150–185) and NS2B (80–95) are either disordered or possibly in conformational equilibrium between folded and disordered states. Studies of the structure and dynamics of the apo DENV2 NS3pro/NS2B as well as its complex with ligands in solution are on-going and will provide important insights in the molecular basis underlying these interactions.

## Supplementary Information

Below is the link to the electronic supplementary material.

Supplementary file1 (PDF 373 KB)
