# Supplementary material for: 1H, 13C and 15N resonance assignment of backbone and IVL-methyl side chain of the S135A mutant NS3pro/NS2B protein of Dengue II virus reveals unique secondary structure features in solution
Source: Biomol NMR Assign. 2022 Feb 12;16(1):135–45. doi: 10.1007/s12104-022-10071-w (PMC9068680; doi:10.1007/s12104-022-10071-w)
Supplement: Supplementary file 1 — Supplementary file1 (PDF 373 KB) [file 12104_2022_10071_MOESM1_ESM.pdf]

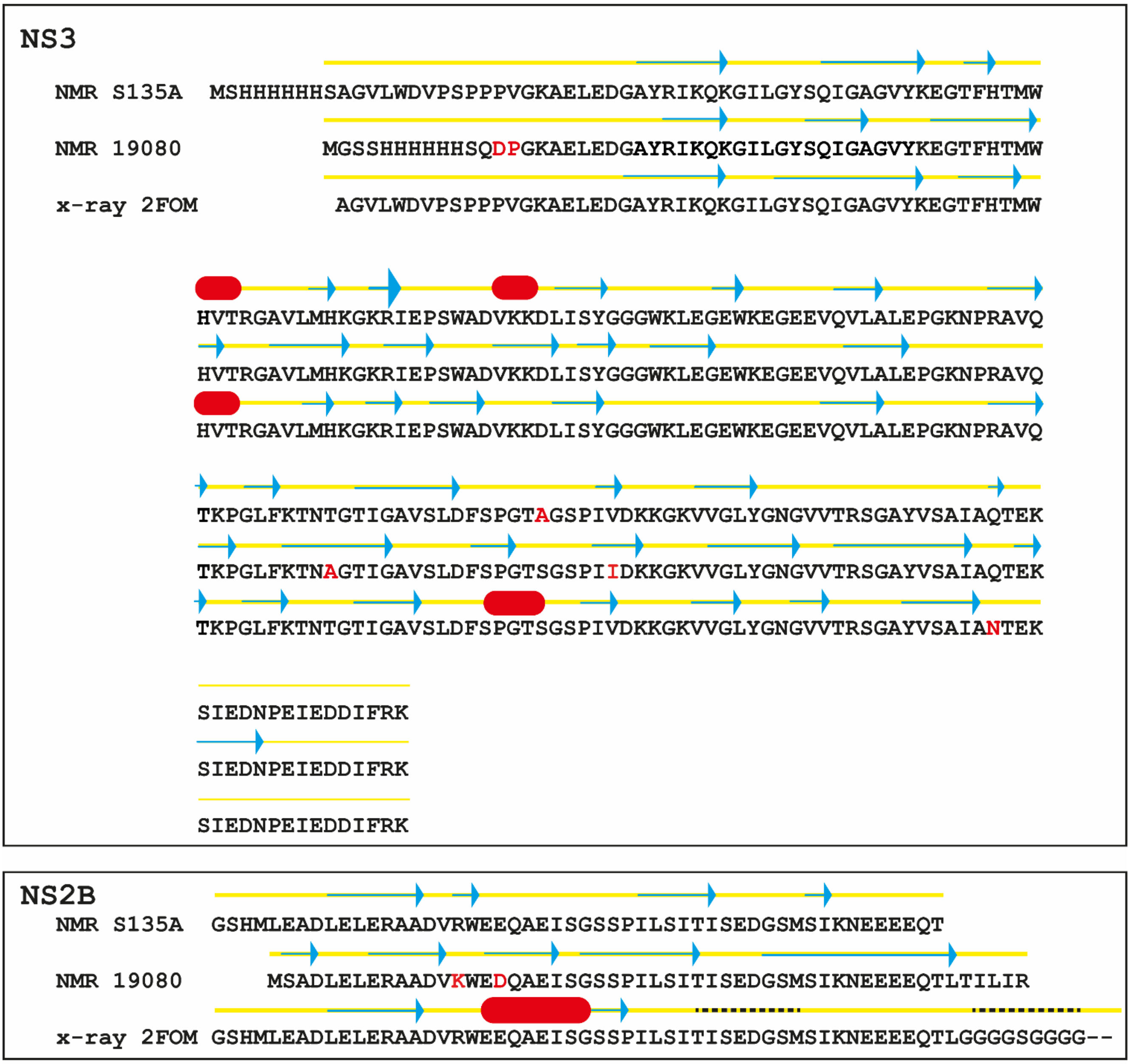

Figure S1. The secondary structure of the DENV2 NS3pro/NS2B does not agree with the secondary structures of previously determined complexes

The secondary structures along the sequence for DENV2 NS3pro (Top Panel) and NS2B (Bottom Panel) predicted by TALOS-N software [40] based on the chemical shifts of the backbone are shown. Top sequence: labelled with NMR S135A, obtained in this study, middle sequence: BMRB 19080 presented earlier in [8] and bottom sequence: PDB: 2FOM x-ray secondary structure extracted according to UCSF Chimera [48]. The yellow lines correspond to loop or coil segments in the protein complex, red squares correspond to α-helices and arrows to β-strands. The sequences are indicated in black, differences in amino acids between sequences are highlighted in red.
